# Enhanced control strategy for photovoltaic emulator operating in continuously changing environmental conditions based on shift methodology

**DOI:** 10.1038/s41598-024-64092-7

**Published:** 2024-06-11

**Authors:** Ambe Harrison, Safeer Ullah, Njimboh Henry Alombah, Mohit Bajaj, Wulfran Fendzi Mbasso, Sheeraz Iqbal, Milkias Berhanu Tuka

**Affiliations:** 1 Department of Electrical and Electronics Engineering, College of Technology (COT), University of Buea, P.O. Box Buea 63, Buea, Cameroon; 2 Quaid-E-Azam College of Engineering and Technology, Sahiwal, Pakistan; 3 Department of Electrical Engineering, Graphic Era (Deemed to Be University), Dehradun, 248002 India; 4 Hourani Center for Applied Scientific Research, Al-Ahliyya Amman University, Amman, Jordan; 5 Graphic Era Hill University, Dehradun, 248002 India; 6 Technology and Applied Sciences Laboratory, U.I.T. of Douala, University of Douala, P.O. Box 8689, Douala, Cameroon; 7 Department of Electrical Engineering, University of Azad Jammu and Kashmir, Muzaffarabad, AJK 13100 Pakistan; 8 Department of Electrical and Computer Engineering, Addis Ababa Science and Technology University, Addis Ababa, Ethiopia; 9 Department of Electrical and Electronics Engineering, College of Technology, University of Bamenda, P.O. Box 39, Bambili, Cameroon

**Keywords:** Solar energy systems, Photovoltaic emulator (PVE), Shift controller (SC), Modified-shift controller (M-SC), Piecewise linear- logarithmic adaptation, Benchmarking test profiles, Energy science and technology, Engineering, Mathematics and computing

## Abstract

This article investigates an inventive methodology for precisely and efficiently controlling photovoltaic emulating (PVE) prototypes, which are employed in the assessment of solar systems. A modification to the Shift controller (SC), which is regarded as a leading PVE controller, is proposed. In addition to efficiency and accuracy, the novel controller places a high emphasis on improving transient performance. The novel piecewise linear-logarithmic adaptation utilized by the Modified-Shift controller (M-SC) enables the controller to linearly adapt to the load burden within a specified operating range. At reduced load resistances, the transient sped of the PVE can be increased through the implementation of this scheme. An exceedingly short settling time of the PVE is ensured by a logarithmic modification of the control action beyond the critical point. In order to analyze the M-SC in the context of PVE control, numerical investigations implemented in MATLAB/Simulink (Version: Simulink 10.4, URL: https://in.mathworks.com/products/simulink.html) were utilized. To assess the effectiveness of the suggested PVE, three benchmarking profiles are presented: eight scenarios involving irradiance/PVE load, continuously varying irradiance/temperature, and rapidly changing loads. These profiles include metrics such as settling time, efficiency, Integral of Absolute Error (IAE), and percentage error (epve). As suggested, the M-SC attains an approximate twofold increase in speed over the conventional SC, according to the findings. This is substantiated by an efficiency increase of 2.2%, an expeditiousness enhancement of 5.65%, and an IAE rise of 5.65%. Based on the results of this research, the new M-SC enables the PVE to experience perpetual dynamic operation enhancement, making it highly suitable for evaluating solar systems in ever-changing environments.

## Introduction

Two critical prerequisites for the implementation of photovoltaic (PV) systems are testing and validation. The utilization of an operational PV system to execute a testing procedure presents numerous complexities, one of which is the inability to enforce a specific environmental profile^1^. Real-time temperature and irradiance conditions are directly reflected in the operation of an actual PV system. It is widely acknowledged that these conditions exhibit continuous variation and are exceedingly complex to forecast, thereby rendering the functioning of photovoltaic systems stochastic. In the realm of solar and renewable energy in general, the requirement for a completely controlled environment to test PV systems becomes an absolute necessity when confronted with this stochastic operation^2,3^. The photovoltaic emulator (PVE) has been emphasized in numerous recent publications as a highly effective tool for simulating the operational characteristics of a real solar panel^4–7^. According to Razman,et. al (2018), a PVE can be defined as a nonlinear power supply that imitates the current–voltage(I-V) characteristics of an actual PV panel^8^. The device provides a flexible operating setting for PV generations systems, with the possibility of manually setting irradiance conditions through a graphical user interface^8,9^.

The PVE has also been solicited in numerous solar energy applications such as the testing of maximum power point tracking algorithms (MPPTs). MPPT is an efficient strategy deployed in the PV system to ensure that the solar panel and the overall system operate at the maximum power point. In the realm of MPPT, several algorithms have been proposed in the literature for optimizing the operation of PV systems^10^. The works of^11^, studied grid synchronization for PV system and implemented a maximum power point tracking framework using the human psychology optimization. In^12^, a learning based hill climbing algorithm is proposed as maximum power point tracking algorithm for a three phase PV system. The authors in^13^, proposed a parabolic curve-fitting based hill climbing algorithm to harvest the maximum power in PV panels operating under dynamic environmental conditions. Several other MPPT algorithms have been suggested in the literature as evident in^14–20^.

A PVE may be conceptualized as a closed loop controlled system, in which the reference of the system is derived by solving the equations of the physical PV model, the plant is powered by a nonlinear power supply, and the system as a whole is regulated by a voltage or current controller that is appropriate^21,22^. Therefore, the aforementioned three subsystems must operate in concert for the PVE to be effective and dependable. In the subsequent subsection, a comprehensive literature review will be presented to situate the present status of PVE.

### Literature review

The classical diode paradigm is an essential component for PVE implementations with respect to reference generation. Literature generally identifies three distinct varieties of diode models: single diode (SDM), double diode (TDM), and three diode (SDM).)^23–25^. These models distinctively vary in accuracy and complexity. Amongst these models, the SDM is highly solicited in renewable energy modelling and especially PV systems due to its ability to balance accuracy and complexity^26^, which are crucial requirements for the implementation of a PVE. After the selection of a diode model, it is imperative that it be seamlessly incorporated into the PVE closed loop system. To accomplish this, model equations must be executed in real time in order to produce a reference signal. Complicated and nonlinear, the defining equations of the PV model are difficult to manipulate^27^. Developing a precise computational handler is therefore one of the primary obstacles to the implementation of a PVE. Within this domain, a number of recent publications have utilized Newton Raphson's algorithm (NRA) to calculate the PV model^28^. In terms of current control, reference generation using the SDM requires the utilization of either the current–voltage (I-V) or the current-resistor (I-R) representation of the SDM^29,30^. The reference generator uses the PVE voltage as input in the former representation while it makes use of the PVE resistance as input in the latter representation, in order to generate a reference current value. The NRA behaves well when implemented with the I-V, but faces several flaws such as oversensitivity and oscillations in the output of the PVE, which are caused by the varying nature of the PVE output voltage^31,32^. The NRA utilizing the I-R representation of the SDM alleviates the aforementioned flaws in the NRA I-V system. Irrespective of changes in PVE voltage or current, the PVE resistance remains constant through its operation, which contributes to stability enhancement of the emulator brought in by the NRA I-R system^31^. Nonetheless, the SDM must be solved and computed through a number of iterations using this type of emulator system. The dynamic response of the overall PVE is influenced by the number of iterations needed to reach a stable computation^33^. This, in turn, can have a series of effects on the efficacy of the emulating system. It is demonstrated that the NRA I-R system can stabilize after undergoing up to 200 iterations under increased PVE burden.

In PVE systems, the iterations constraint continues to be a significant factor^34^. This is due to the fact that restricting the number of iterations provides an appealing guarantee of enhanced performance for such systems. Reference generation in PVE through linearization is able to bridge the gap of iterations, as evidenced in the four segments^35,36^ and 12 segments linearization^6,7^ computation strategy. Linearization approaches, in their pursuit of simplicity, undermine the accuracy of the PVE, thereby constituting its primary drawback. Furthermore, the intricacy of the system becomes more significant as the quantity of segments necessary to execute the linearization strategy increases. The 12 segment linearization method, for instance, necessitates the memorization of I-V curve segments by a neural network model. For real-time operations, computationally implementing such a model in a microprocessor remains extremely difficult. In PVE systems, binary search (BS) computation has recently emerged as a potent numerical handler^8,31^. The simplified solver nature of the BS, in contrast to the NRA, precisely satisfies the simplicity criteria for PVEs. Additionally, the BS attains a higher level of accuracy in comparison to the Linearization method due to its capability of processing the nonlinear equations of the SDM without requiring linearization. It was demonstrated that the PVE can undergo a maximum of twenty iterations when the BS is combined with the I-R representation of the SDM ^31^. Unavoidably, this reduction in the number of iterations improves the dynamic capabilities of the PVE. Due to these characteristics, the BS I-R emulator is a straightforward yet potent system.

**Figure 1:**
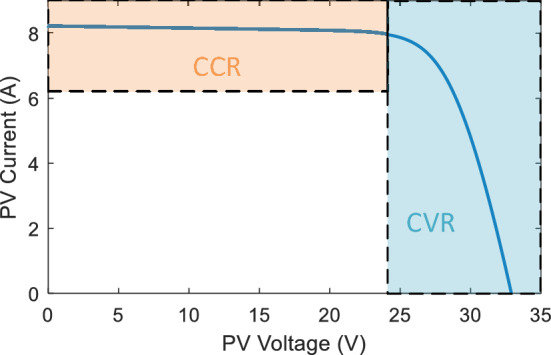
I-V curve of the PV highlighting CCR and CVR.

Regulation and control are critical components of the close loop PVE system in order to ensure that an emulator operates reliably. The controller possesses the capability to establish the necessary constant and transient responses of the PVE, contingent upon the reasonable design of the nonlinear power plant^37,38^. Several modern PVEs have incorporated the PI controller extensively during the adjustment phase^39–41^. As a result of the PVE plant's intrinsic nonlinearity, the PI controller cannot consistently ensure a satisfactory dynamic response. Moreover, its robustness could diminish under extreme operating conditions, such as those characterized by substantial fluctuations in the PVE burden and ever-changing environmental conditions. An additional critical aspect concerning the functioning of PVE pertains to the feasibility of the controller in relation to the segments of the I-V curve. As shown in Fig. 1, the I-V curve can be partitioned into a constant current region (CCR) and a constant voltage region (CVR). The output of the PVE may exhibit oscillatory behavior in either region for specific categories of controllers. Consequently, the PVE output oscillates in the CVR of the PVE when a current controller is employed. A minor variation in voltage yields a significant alteration in current within this particular region. Conversely, the utilization of a voltage controller induces oscillations in the PVE of the CCR, as even a marginal alteration in the current results in an exponential surge in the voltage. This reference signal inconsistency presents a significant obstacle to the operation of directly controlled PVEs. Hybrid controlled strategies are suggested as potential remedies for the aforementioned issue. Based on the region of operation of the PVE, this strategy employs and activates two controllers: a voltage-PI controller is utilized in the CVR, whereas a current-PI controller is implemented in the CCR. This approach enhances the dynamic performance of the PVE, particularly in the vicinity of the knee point of the curve. However, it necessitates the implementation of two controllers, thereby increasing the complexity of the control algorithm.

Among several PVE controllers in the literature, fuzzy logic control (FLC) has been employed to improve the performance of PVEs^42^. However, the primary limitations of the FLC in the context of PVE are its heavy reliance on the expertise of the designer and its complex fuzzy principles. Concerns regarding instability in the PV were resolved by suggesting an enhanced FLC^43^. A fuzzy logic law is utilized in this control strategy to rectify the difference between the reference current and the actual PVE current. The resulting value is then transmitted to a PI controller. On a 255W PV system, the overall PVE was implemented and evaluated; it was discovered that its transient performance was considerably superior to that of the traditional PI controller. Within the domain of control, an approach involving a damping injection controller was suggested as a means to alleviate the speed and accuracy constraints of prior emulators. The energetic-based controller is specifically engineered to mitigate the nonlinearity inherent in the PVE plant, while also addressing concerns pertaining to the operational region of the PVE, namely the CCR and CVR^44^. For CVR operation, a damping injection voltage controller is synthesised, whereas in the CCR, a damping injection current controller is employed to stabilise the PVE current. In addition, an overlap is implemented around the MPP by the control algorithm in order to prevent switching issues caused by measurement noise. The authors performed a number of simulations in order to demonstrate the controller's applicability to various PV configurations. One of the primary benefits of this controller in comparison to the PI controller is its comparatively greater bandwidth. However, the implementation of the algorithm necessitates the adherence to two complex nonlinear control laws for each region of the PV, thereby adding complexity to the overall PVE. Furthermore, the functionality of the controller is heavily reliant on parameters of the PV plant that are susceptible to fluctuations from their designated values.

In terms of nonlinear power supply, several topologies of DC-DC converters have been utilized in the realization of PVEs^45–48^. Buck converters, a subset of switch mode power supplies, are renowned for their adaptability and user-friendliness. Additionally, it permits the PVE to function across a substantial portion of the I-V range, given that the input voltage exceeds the open circuit voltage of the PV system^4^.

A high bandwidth controller was developed to emulate the characteristics of 60W PV module using backstepping methodology^7^. Backstepping, which is classified as a nonlinear controller, is highly suitable for mitigating nonlinearity in PVE systems. Implementation complexity and reliance on both the model parameters and the complete conditions of the systems are, however, significant drawbacks of this PVE. In order to increase the PVE's applicability, it is critical to restrict its complexity. Furthermore, the development of controllers that rely minimally or not at all on model parameters offers opportunities to enhance the functionality of PVEs. Additionally, it is preferable to restrict the necessary system states for the implementation of the controller in order to prevent measurement noise from rendering the control ineffectual in real-world scenarios. Razman et al. (2020) proposed a novel controller known as "shift" control within the domain of this spirit^8^. It is derived from the FLC heuristic and is extended through the use of straightforward linear logic. As a result, neither additional imprecise rules nor designer expertise are necessary to implement the algorithm. The primary characteristics of the controller are its ease of use and speed. As a result, it possesses the capability to swiftly emulate a real photovoltaic panel, surpassing the performance of contemporary controllers like PI and FLC.

**Figure 2:**
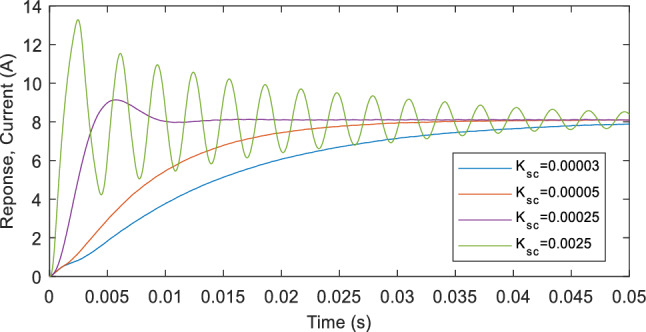
Step response of the PVE with shift controller close loop system at different values of ${K}_{sc}$.

The shift controller uses a single gain parameter, ${K}_{sc}$ to regulate the PVE output through adjustment of the discrepancy between the PVE current and the reference via a linear control law. Due to its over simplicity, the performance of the controller is highly contingent upon the PVE load. However, the conventional shift controller (C-SC) does not integrates this consideration. As a consequence, the dynamic performance of the C-SC depreciates with increase in the load. In addition, the control method suffers from a trade-off between transient speed and steady states oscillations. As demonstrated in Fig. 2, increasing the value of ${K}_{sc}$ makes the closed loop PVE system faster with a small transient time. However, this is achieved at the expense of oscillations in steady state. Conversely, reducing the gain parameter improves the steady state of the system at the expense of a slow transient response. Therefore based on this observation, ${K}_{sc}$ in the C-SC must be chosen on the basis of trade-off. For the step-response illustration in Fig. 2, this corresponds to a value of 0.0005. Following this consideration, the efficacy of the PVE will remain moderate and far from optimality. The performance of the controller and the PVE will degrade for increasing load as the settling time will grow to a significant value. Such a degradation inevitably deprecates the efficiency of the emulator.

### Motivation

This investigation was undertaken due to various compelling considerations. The primary objective of this study is to create a PVE that alleviates the constraints of current emulators. After conducting thorough preliminary research, it was determined that PVE based on Binary Search (BS) using the current-resistor (I-R) representation of the single diode model (SDM) and regulated by the shift controller is a promising option. The BS, known for its feature of restricting the number of iterations, was recognized as the primary computational tool for PVE. Furthermore, the shift controller was identified as one of the most promising controllers for PVE applications. However, the limitation of the traditional shift controller that was mentioned earlier is the main focus of this study. The C-SC's dynamic response is greatly influenced by the PVE load, while its control law remains unaffected by it. Therefore, under heavy load conditions, the settling time of the PVE increases to a considerably greater extent, resulting in a direct decrease in the system's emulation efficiency. Hence, it was crucial to adapt the C-SC to accommodate both the output and fluctuations in the load. As a hypothesis, implementing a modification that establishes a correlation between the control law and the PVE load could enhance the PVE transient response and improve its overall efficiency.

### Main contribution

To address the deficiencies highlighted in the aforementioned literature review, this study introduces a novel solar panel emulator. By utilizing BS computation and an I-R representation of the SDM, the proposed prototype is equipped with a buck power supply that is regulated by a modified-shift controller (M-SC). This paper centers on the improvement of the conventional shift controller (C-SC) within the domain of PVE. In this investigation, the first modification to the SC is suggested. The modified controller under consideration aims to establish a correlation between the control law and the PVE output to guarantee a more rapid dynamic response and optimal emulation efficiency, even under heavy load. To modify the controller law and enhance the performance of the PVE, we consequently suggested a new piecewise linear-logarithmic adaptation function. Therefore, the controller is adjusted within a specific operational range using a linear function of the PVE load. In contrast, in other regions, an adaptation is implemented using a logarithmic function. The modified controller that is proposed attains substantial improvements in both dynamic response and efficiency, while maintaining the integrity of the overall control law. A novel methodology is employed to implement the entire PVE, which is subsequently verified via numerical investigations. Several testing profiles are suggested for the purpose of comparing the proposed PVEs. By means of a succession of comparative analyses, the proposed controller consistently establishes itself as a substantially enhanced version of the traditional SC. Moreover, it is exceptionally well-suited for the evaluation of photovoltaic panels in conditions of fluctuating temperature and irradiance. Hence, the principal contribution of this study can be succinctly outlined as follows:

1. The first modification to the SC is proposed
2. A piecewise linear-logarithmic adaptation scheme is proposed to enhance the C-SC
3. A unique methodology considering three global testing patterns (8-consistent Irradiance and Temperature, fast changing load, and continually changing irradiance and temperature) is proposed to benchmark the PVE
4. Proposed PVE perpetually enjoys high dynamic response, reduced integral of absolute error and increased efficiency

### Paper Methodology and organization

**Figure 3:**
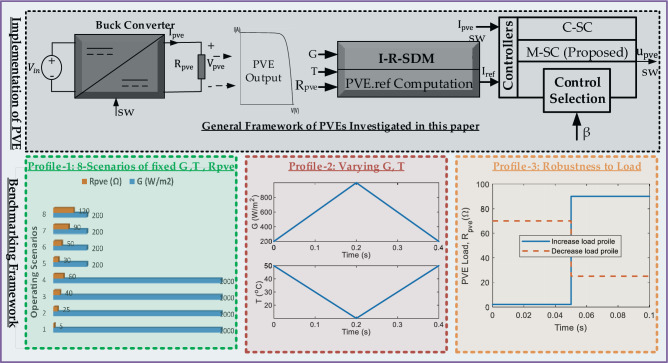
Graphical methodology adopted by this study.

A novel methodology is utilized in this article to implement and benchmark the proposed PVE. As illustrated in Fig. 3, a numerical simulation platform is built using the MATLAB/Simulink software (Version: Simulink 10.4, URL: https://in.mathworks.com/products/simulink.html). The neutral platform on which the C-SC and proposed M-SC are executed permits the selection of a particular controller via the transmission of the command β. The C-SC is enabled on the simulation platform when β equals zero, whereas the M-SC is enabled when β equals one. Every other element of the PVEs is maintained consistently. A common I-R SDM is computed online using the BS computation. A common buck converter is adopted as the switched mode power supply (SMPS). Three benchmarking profiles are taken into consideration during the dynamic testing and validation phase, as detailed below.

- *Profiile-1*: In this profile, 8 different scenarios of consistent Irradiance (G), Temperature (T) and PVE load ${\text{R}}_{\text{pve}}$ are adopted as shown in Fig. 3
- *Profile-2*: A continuously changing pattern of G, and T is adopted. In this profile G ranges from 200 to 1000W/m^2^ while T ranges from 10 to 50 °C, as shown in Fig. 3.
- *Profile-3*: In this profile, a test of robustness is conducted under increasing and decreasing load as shown in Fig. 3

## Photovoltaic emulator system modeling

**Figure 4:**
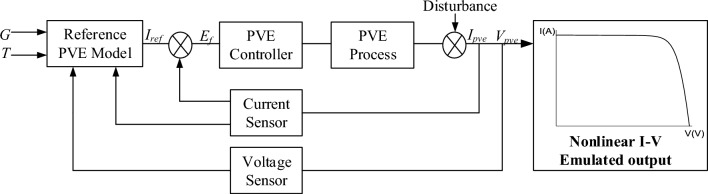
Close loop model of the PVE.

A PVE can be explicitly seen as an interconnected system made up of a reference model, a PVE controller and the system process. The reference model, derived using a model of the solar panel under emulation receives two inputs temperature (T) and Irradiance (G), which are externally provided by the system user. A nonlinear power stage (DC-DC converter), usually adopted as the process or plant, is regulated by the PVE controller to emulate the behavior of the solar panel. The close loop system model of the PVE can be seen in Fig. 4. It can be seen that unlike traditional control systems, the reference model depends on the output and forms an external close loop system in the PVE. Thus for reliable operation of the PVE, respective subsystems must be designed considering the synergic effect of the neighboring systems. This section is aimed at designing the model components of the PVE for an accurate operation.

### PV model and integration into PVE

Being a key element of the PVE, the PV model has a major contribution to the accuracy and efficiency of a PVE. Similar to contemporary studies, the single diode model (SDM) is considered in this paper for its ability to balance complexity and accuracy. The foundational equations of the SDM are presented in Eq. (1–3), and supported by^49^. The term ${I}_{pv}(G,T)$ denotes the PV output current and its functional dependence on G and T

1 $${I}_{pv}(G,T)={I}_{ph}-{I}_{s}\left[\mathit{exp}\left(\frac{\left({V}_{pv}+{I}_{pv}{R}_{s}\right)}{n{N}_{s}{K}_{B}T/q}\right)-1\right]-\frac{{V}_{pv}+{I}_{pv}{R}_{s}}{{R}_{sh}}$$

2 $${I}_{ph}=\frac{G}{1000}({I}_{ph(ref) }+{K}_{i}(T-{T}_{ref})$$

3 $${I}_{s}={I}_{s(ref)}{\left(\frac{T}{{T}_{ref}}\right)}^{3}exp\left[\frac{q}{k}\left(\frac{{E}_{g,ref}}{{T}_{ref}}-\frac{{E}_{g}}{T}\right)\right], {E}_{g}={E}_{g(ref)}(1-0.000267(T-{T}_{ref})$$

4 $${R}_{sh}={R}_{sh,ref}\left(\frac{1000}{G}\right)$$

where ${I}_{ph}$, ${I}_{s}$, ${V}_{pv}$, ${N}_{s}$, ${K}_{B}$, $q,$ ${E}_{g}$, denote the model photocurrent, reverse saturation current, output voltage, number of model cells in series, Boltzmann constant ($1.381\times {10}^{-23}J/K)$, charge constant ($1.602\times {10}^{-19}C)$, and energy of band gap respectively. The subscript (ref), denotes a specific parameter at standard test conditions (STC), i.e. $G=1000W/{m}^{2}$ and $T=25^\circ{\rm C}$. The implementation of the SDM based on the above equations, requires some parameters which can only be obtained from the manufacturer catalogue. This is the case with the parameter ${K}_{i}$, known as the temperature coefficient of short-circuit current. Furthermore, some other parameters not provided by the manufacturer have to be identified. These are shunt resistance ${R}_{sh},$ series resistance ${R}_{s}$, ${I}_{ph}, n$ and ${I}_{s}$. Extensive contemporary works has been covered in literature on parameter identification of the PV module^26,50–52^, thus this study will not emphasize further on that. Throughout this study, a 200W solar module of type KC200GT is adopted^53^. From the datasheet the parameter ${K}_{i}$ has the value of 0.00318 A/^o^C. The module has open circuit voltage of 32.9 V and short-circuit current of 8.21A.

The above equations model the I-V representation of the SDM. When the model of the PV is established, it has to be integrate into the PVE. The integration of the PV model requires online numerical resolution of the prescribed equations. In this realm, Newton Raphson Algorithm (NRA) is traditionally used for this purpose. However, in PVE setting, utilizing the I-V representation of the SDM sources oscillations in the emulator output. To overcome this, the I-R model is adopted. This representation receives as input the PV resistance computed as ($${V}_{pv}/Ipv )$$ to generate the reference current. Thus the input of the PVE is always stable despite variations in the PVE voltage or current. As evidenced in^31^, the NRA resolution of the I-R model is iteratively inefficient. It can take up to 200 iterations to generate a stable output. This increased count of iterations inevitably deprecates the dynamic response and consequently the efficiency of the PVE.

The Binary search (BS) computation, a simple yet powerful computational algorithm is suited for handling the I-R model of the PV at a reduced oscillation count^32,39^. It has been shown this computational strategy can limit the iteration count in the PVE to a max of 20 iterations^31^. For the current investigation undertaken in this paper, the BS is thus consistently adopted for reference generation.

5 $${I}_{pv}\left(G,T,{R}_{pv}\right)={I}_{ph}-{I}_{s}\left[\mathit{exp}\left(\frac{{I}_{pv}\left({R}_{pv}+{R}_{s}\right)}{\frac{n{N}_{s}{K}_{B}T}{q}}\right)-1\right]-\frac{{I}_{pv}\left({R}_{pv}+{R}_{s}\right)}{{R}_{sh}}$$

6 $${H}_{pve}(G,T,{R}_{pv})={I}_{ph}-{I}_{s}\left[\mathit{exp}\left(\frac{{I}_{pv}\left({R}_{pv}+{R}_{s}\right)}{\frac{n{N}_{s}{K}_{B}T}{q}}\right)-1\right]-\frac{{I}_{pv}\left({R}_{pv}+{R}_{s}\right)}{{R}_{sh}}-{I}_{pv}$$

7 $${I}_{pv}={I}_{pv(\text{min})}+\left(\frac{{I}_{pv\left(\text{max}\right)}-{I}_{pv\left(\text{min}\right)}}{2}\right)$$

**Figure 5:**
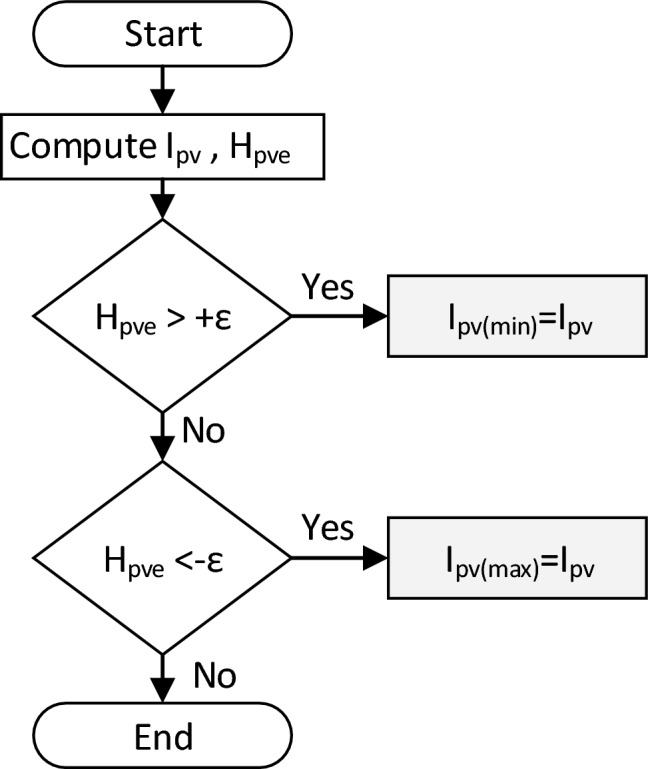
Block diagram of the Binary search (BS) computation.

**Figure 6:**
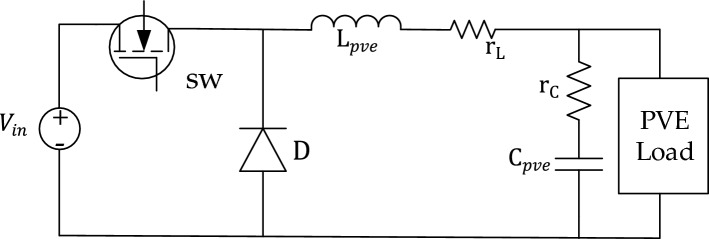
Buck DC-DC Converter electrical diagram and its designed values.

Thus the BS computation is used to resolve Eq. (1), wherein the latter goes through a transformation to form an I-R model as shown in Eq. (5). Subsequently the BS algorithm as described in Fig. 5 is used to solve the Eq. (6) in order to generate the required reference current. In the computation process, the maximum ${I}_{pv(max)}$ and minimum values ${I}_{pv(min)}$ are adopted iteratively using Eq. (7) such that the function ${H}_{pve}$ is minimized. The computation is stops when the following condition is fulfilled (Fig. 6):

8 $${-\varepsilon <H}_{pve}<+\varepsilon$$

where $\varepsilon$ is small error tolerance, and is assigned a value of 0.001A.

### PVE power stage design

In order to capture the static as well dynamic characteristics of an actual PV, a nonlinear power supply is required. As announced earlier, this study consistently adopts a Buck DC-DC converter in its power stage. Ease of controllability is one of the special feature of this converter. In addition, the converter is efficient and has a lower count of components, which profits the emulator^8^. The converter is characterized by its major components namely inductance ${L}_{pve}$, and Capacitance ${C}_{pve}.$ Therefore proper operation of an emulator demands a proper design of these components. Furthermore. For more practicality parasitic components are introduced into the components namely ${r}_{L}$ and ${r}_{C}$

The converter is consistently designed in accordance to the guide provided in^54^. The key consideration in the design is to ensure that the input voltage is sufficiently higher than the open circuit voltage of the system to allow the converter sweep over a considerable large region of the I-V curve. Furthermore, the ripple in the output of the PVE is limited to 1%. The design maximum operation irradiance of the PVE is $1000W/{m}^{2}$ and minimum irradiance of $200W/{m}^{2}$. Based on this premise, the designed values of the converter are obtained as presented in Table 1

**Table 1:** The designed values of the converter.

Parameter	Value
Input voltage, V_i_	48 V
Switching frequency, f_s_	31 kHz
Duty cycle, D	0.05–0.9
Internal resistance of L, r_L_	0.631 Ω
Internal resistance of C, r_C_	0.134 Ω
PVE inductance, L_pve_	3.52 mH
PVE Capacitance, C_pve_	80 uF

### PVE controller design based on shift control algorithm

Conventionally proposed by Razman Ayop and his colleagues in 2020, the Shift controller (SC) is a simplified control strategy that is inspired by fuzzy-logic heuristics. It is based on the relationship between system discrepancy and control action. The SC was developed to provide simplified and rapid control of the buck converter system. Thus for this class of system, the discrepancy and control action (change in duty cycle) observes a linear relationship with upward and downward shifts, which can be approximated using straight lines. If the discrepancy between the plant output and the desired reference is considered as ${E}_{f}$ and the control action written as $dD$ then, the linear relationship can be approximated by Eq. (9):

9 $$dD={E}_{f}+d{E}_{f}, d{E}_{f}={E}_{f(i)}-{E}_{f(i-1)}$$

where $d{E}_{f}$ is the change in error between a previous $(i-1)$ and current instant $(i)$. Therefore, the controller is consistently implemented with a memory function to hold previous computations.

It can be observed that Eq. (9) is the equation of a line with intercept $d{E}_{f}$. The linear relationship between the error and the duty cycle is derived from the heuristics in Table 2. Because the relationship between the two variables is not static, a compensation gain, m is introduced so that Eq. (9) becomes Eq. (10). This imply that the speed of the controller, notably its transient response is controlled by m. At low values of m, the system has a slow response, while if the value of m becomes too large, the close loop system may become unstable due to oscillations. To circumvent the need of adjusting m, the intercept of the control line is manipulated so that initially when the system is its transient, the intercept is shifted upward, while in the steady-state, it becomes small and the intercept shift downwards. This is easily detected from the changes in the error.

**Table 2:** Heuristics of the shift controller.

${{\varvec{E}}}_{{\varvec{f}}}$	Negative small	Negative large	Zero	Positive small	Positive large
${\varvec{d}}{\varvec{D}}$	Negative small	Negative large	Zero	Positive small	Positive large

10 $$dD={mE}_{f}+d{E}_{f}$$

Considering m = 1, and the relevant substitution of ${E}_{f}$, Eq. (10) undergoes a transformation to:

11 $$dD={2E}_{f(i)}-{E}_{f(i-1)}$$

The current control duty cycle can thus be derived from the following Equations:

12 $${D}_{(i)}={D}_{(i-1)}+dD$$

13 $${D}_{(i)}={D}_{(i-1)}+\frac{{K}_{sc}}{Ref}({2E}_{f\left(i\right)}-{E}_{f\left(i-1\right)})$$

where ${K}_{sc}$ is the new shift controller gain, and ref is the reference signal which is generally tuned manually using trial and error method and found optimal at the value wherein the controller obeys design criteria. The optimal value of ${K}_{sc}$ that observes the criteria of settling time of 30 ms, at the minimum operating load of 2Ω, is found to have a value of 0.00005. The ref in introduced in the control law to correlate the control action with the operation of the PVE, notably irradiance and temperature. Thus the control law is adaptive with respect to changes in environmental settings. It is must be noted that the reference signal is determined from environmental settings G and T. Based on th above development, it is evident that the SC is a simplified control algorithm requiring very little computational resources when implemented in a processor.

**Figure 7:**
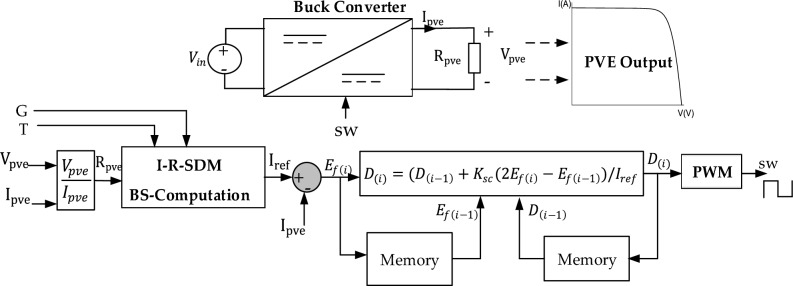
Integration of the BS computation and the SC into a PVE.

The integrated SC algorithm for a PVE is graphically described in Fig. 7. In the PVE context, two external inputs are provided by the operator, notable G and T. The output current and voltage of the PVE ${I}_{pve}, {V}_{pve}$ are sensed and used as feedback into the system. Considering the I-R model, the PVE resistance ${R}_{pve}$, is obtained from the digital division of the PVE voltage and current. The reference current is generated by the I-R-SDM block, which involves the BS computation of the I-R SDM. Finally, the control action is generated according to Eq. (13), and converted into a PWM of frequency ${f}_{s}$ used to adjust the buck converter.

#### Limitation of the conventional shift controller (C_SC)

It cannot be further emphasize that the SC is a very simplified yet powerful control strategy. Nevertheless, it has some shortcomings worth addressing. These shortcomings are summarized under two points stated below:

##### *Problem-1*

Tradeoff between transient response (speed) and steady-state oscillations.

As previously stated, the compensation gain significantly influences the behavior of the shift controller system that operates in a closed-loop configuration. A consequence of this gain is a compromise between response speed and stead-state oscillations. When the initial gain is substantial, the system exhibits a gradual response and reaches a stable state. Conversely, a substantial value of the gain expedites the system while sacrificing steady-state oscillations. Therefore, it is necessary to contemplate a trade-off when determining a secure value for the gain. The crux of the matter is that this compromise undermines the controller's overall efficacy. The correlation between the gain and the system's response is illustrated in Fig. 1.

##### *Problem-2*

No correlation between the control law and the output of the PVE.

**Figure 8:**
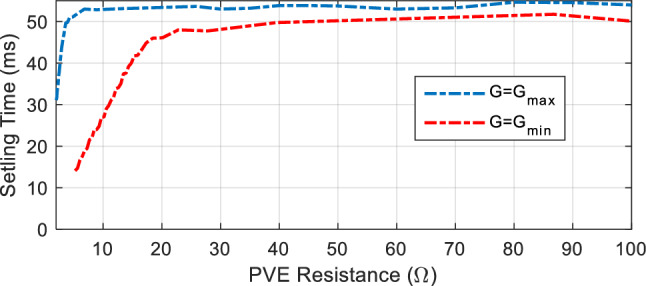
Close loop step response of the PVE, highlighting the effect of load on setting time.

A more significant deficiency of C-SC is the absence of correlation between the control action and the PVE output. As shown in Eq. (13), the PVE load ${R}_{pve}$ is not accounted for in the C-SC control law. This is analogous to the operation of a solar panel under a specific load condition or the design of a PVE to operate at a fixed load. Given that actual systems are susceptible to operating at varying loads, this functionality must be accounted for by the PVE's controller. This can be achieved through the implementation of load-dependent controller adaptability. As depicted in Fig. 8, the effect of the PVE load, denoted by ${R}_{pve}$, indicates that the settling time of the PVE varies substantially with load and becomes substantially larger at high load. This emphasizes the necessity of adjusting the controller in response to the generated demand.

## Proposed modified shift controller

**Figure 9:**
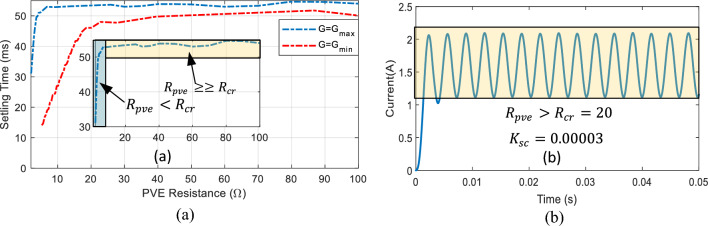
(**a**) Close loop step response of PVE illustrating critical boundary. (**b**) Step response of PVE showing oscillations at steady state for PVE resistance above critical boundary, with large value of compensation.

Triggered by the two problems outlined, we proposed the first modification of the C-SC. The new controller termed the modified shift controller (M-SC) seeks to solve the stated problems. Thus we must resort to an adaptive shift controller. Following this, the control law in Eq. (13) is modified to:

14 $${D}_{(i)}={D}_{(i-1)}+\frac{{K}_{sc}}{Ref}{R}_{M}({2E}_{f\left(i\right)}-{E}_{f\left(i-1\right)})$$

where ${R}_{M}$ is a function of the PVE resistance written as ${R}_{M}({R}_{pve})$, introduced to adapt the control action. Based on observation, it can be seen in Fig. 9a that the settling of the PVE increases consistently up to a critical value of PVE resistance ${R}_{cr}$. Above this resistance, the settling time increases slowly. This explicitly mean that the control action must be made larger at loads lower than ${R}_{cr}$, as well as properly managed for loads greater than the critical condition to avoid oscillations in steady-state. Therefore, it was important to do an extra adjustment of the control law following current state of load. Since the minimum load of a PVE is obtained at the maximum irradiance ($G={G}_{max}$), it is sufficient and necessary to establish the adaptive design based on this condition. We proposed the following piecewise linear-logarithmic function to create the descried adaptation.

15 $${R}_{M}=\left\{\begin{array}{c}{a\times R}_{pve}, {R}_{pve}<{R}_{cr}\\ {\text{b}\times \text{log}}_{10}({R}_{pve}), {R}_{pve}\ge {R}_{cr}\end{array}\right.$$

where a and b are positive constants designed offline to perfect the adaptation scheme. The equation defines that, below ${R}_{cr}$, the adaptive function and consequently the control action increases linearly with the PVE resistance. It must be noted that as the resistance increases, the net value of the compensation gain that is ${K}_{sc}{R}_{M}$ increases sufficiently enough to enhance the system transient response up to ${R}_{cr}$. If this large value of compensation is maintain above ${R}_{cr}$, the control action would make the system oscillate periodically at steady state as observed in Fig. 9b

Thus it is imperative to develop a second relation that manages the control action for higher loads. We propose a logarithmic function for this purpose as seen in Eq. (15). This ensures that the response of the PVE is oscillation free irrespective of the load conditions. The value of a and b are chosen offline as a = 1 and b = 2, while ${R}_{cr}=10\Omega .$ The gain ${K}_{sc}$ is chosen as $0.00003$ to be slightly lower than a similar gain for the C-SC (0.00005). Therefore, the proposed adaptation would enhance the response, accuracy as well as efficiency of the PVE.

**Figure 10:**
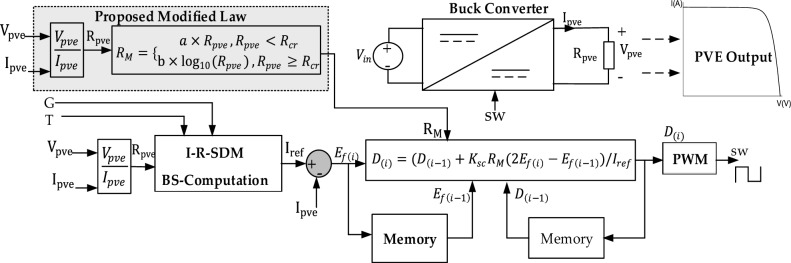
Implementation of the proposed modified shift controller.

The implementation of the M-SC in a PVE setting is shown in Fig. 10. It is evident that the new controller maintains the simplicity of the original C-SC. The novel addition lies in the computation of the adaptive function ${R}_{m}$, which is also a simple function and does not rely on extra sensor measurement.

## Results and discussions

As illustrated by the graphical methodology in Fig. 3, the PVE system as a whole is executed using MATLAB/Simulink (Version: Simulink 10.4, URL: https://in.mathworks.com/products/simulink.html). A number of numerical simulations are conducted in order to assess the efficacy of the proposed controller relative to the conventional controller. As depicted in Fig. 6, the controllers are implemented on a neutral platform with shared system parameters. Furthermore, the subsequent performance indicators are taken into account:

- *Percentage Error*, used to assess the steady-state (only) performance of the PVE, see Eq. (16)
-*Integral of Absolute Error (IAE):* Used to quantify the effectives of the controller in terms of steady-state accuracy and dynamic response. It is computed using Eq. (16)
- *Emulation efficiency*, calculated with reference to the actual PV solar panel, using Eq. (17)
- *Settling time* ${T}_{s}$, recorded as the time required by the PVE to reach and stay within 2% of its desired output
  16 $${e}_{pve}=\frac{\left|{I}_{pve}-{I}_{pv}\right|}{{I}_{pv}}\times 10$$
  17 $$IA{E}_{pve}={\int }_{o}^{t}\left|{I}_{pve}-{I}_{pv}\right|dt$$
  18 $$\eta =\frac{{\int }_{o}^{t}{P}_{pve}(t)dt}{{\int }_{o}^{t}{P}_{pv}\left(t\right)dt}$$

The PVE is expected to mirror the actual solar panel in a specific duration of time, thus it is crucial to limit the emulation time of the PVE. Therefore, an emulation time constraint is adopted throughout the simulation as defined in Eq. (20). The duty cycle control action is also constrained according to Eq. (19), with maximum and minimum value presented in Fig. 6

19 $$0.05\le D\le 0.9$$

20 $${T}_{pve}\le 50ms$$

Two classes of simulations are performed as discussed in details in the following subsection.

### Steady state evaluation of the PVE

**Figure 11:**
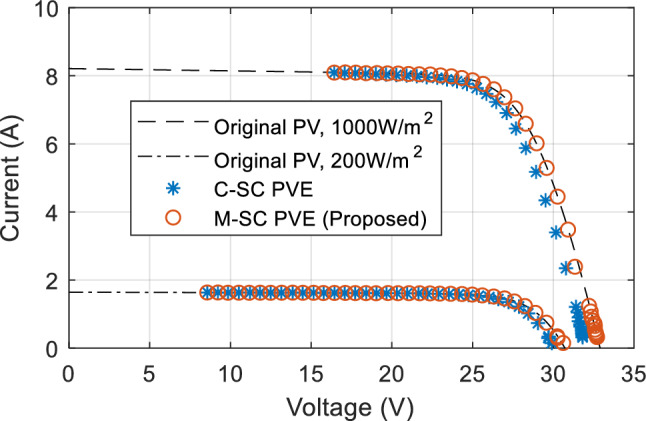
Steady state results of the PVEs: Emulated data superimposed on I-V curve of actual panel.

This evaluation seeks to appraise the steady-state performance of the proposed PVE measured by percentage error. The PVE systems are coupled with varying load values from 2Ω to 200Ω, their output constrained by the simulation time is recorded as current and voltage. The emulated data of the PVEs superimposed on the actual panel I-V curve is shown in Fig. 11. It can be seen that all the PVEs demonstrate the ability to mirror the actual PV I-V curve. Both PVEs do not cover the entire I-V curve due to the control constraint imposed in the PVE. This constraint prevents the buck converter from operating at extreme duty cycle in order to prevent the switching devices from switching stress. Following that the PVE is said to operate between a minimum ${R}_{pve}$ of 2Ω and a maximum value of up to 200Ω.

**Figure 12:**
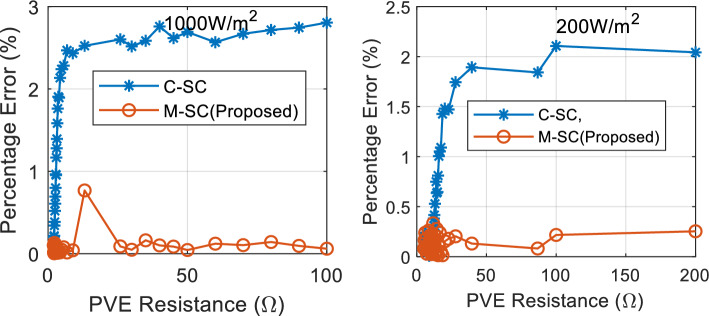
Steady state results of the PVE. (Left) ${e}_{pve}$, $G={G}_{max}$(Right) ${e}_{pve} , G={G}_{min}$.

**Figure 13:**
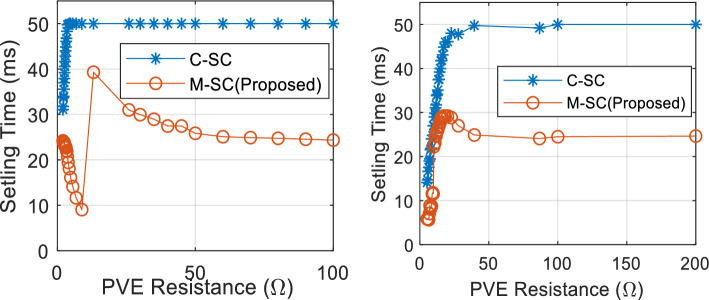
Steady state results of the PVE. (Left) ${T}_{s}$, $G={G}_{max}$(Right) ${T}_{s} , G={G}_{min}$.

It can be seen that in Fig. 11 that the proposed PVE is more consistent with the actual solar panel as compared to the C-SC. The quantitative performance of the PVEs are showcased in terms of accuracy measured by ${e}_{pve}$ and settling time in Figs. 12 and 13 respectively. The proposed M-SC records a maximum ${e}_{pve}$ of 0.7677% as compared to 2.805% by the C-SC at $G={G}_{max}=1000 W/{m}^{2}$. This represent an approximately 2% improvement in accuracy. Additionally, at the minimum irradiance level, the proposed M-SC records a maximum of 0.3337% as compared to 2.106% recorded by the C-SC. Based on these observations, the M-SC achieves a better accuracy. It can be seen in Fig. 13(left) and (right) that the settling time ${t}_{s}$ of the C-SC increases in an approximately linear order up to around 10Ω, while that of the M-SC decreases consistently. The decreasing settling time of the proposed PVE in this region is contributed by the adaptation mechanism introduced into the control law. The increasing setting of the C-SC on the other hand contributes to its low accuracy as evidenced in Fig. 12(left). It can also be confirmed that ${e}_{pve}$ of C-SC increases consistently as seen in Fig. 12(left) and (right). At higher resistance as it is cased for ${R}_{pve}>10\Omega$, the C-SC PVE records a higher settling time above 50 ms. However, due to emulation constraint, the settling time is limited to 50 ms. Conversely, the M-SC consistently reduces the settling time. For instance, at $G={G}_{max}$, the M-SC reduces the settling time from 39.3 ms at 13.11Ω to 24.35 ms at 100Ω (see Fig. 13(left)). Similarly, the settling is reduced to 24.35 ms at the minimum irradiance. This shows that the adaptive mechanism handles the problems of the C-SC. In the subsequent sections of this paper, the dynamic response of the PVEs will be evaluated under different settings of G and T.

### Dynamic evaluation of the PVE

This section aims at assessing the dynamic response of the PVE considering diverse operating conditions such as changing G, T, as well as changing load. Three dynamic test profiles are used to appraise the performance of the PVE.

#### *Test profile 1- Simulation under 8 scenarios of G-R*_*pve*_

**Figure 14:**
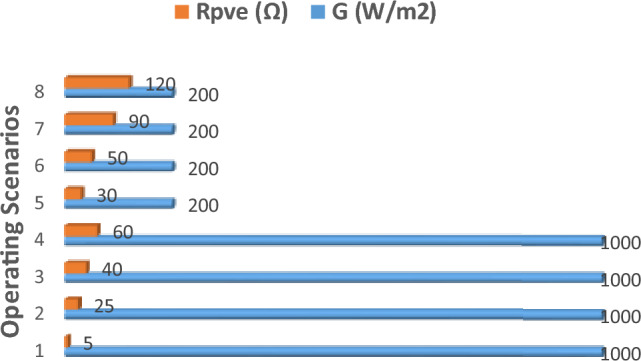
Different operating scenarios in Test profile 1.

In this test, the PVEs are subjected to distinctive settings of G and ${R}_{pve}$. A total of 8 scenarios are used as shown in Fig. 14. Throughout this scenarios, the temperature is held constant at $T=25^\circ{\rm C}$. The values of G, and ${R}_{pve}$ settings at the different scenarios is tabulated in Table 3.

**Table 3:** Values of G, R_pve_ settings in test profile-1 with quantitative result summary.

Operating conditions, T = 25			PVE settling time, t_s_ (ms)		IAE	
G (1000W/m^2^)		R_pve_ (Ω)	C-SC	M-SC (Proposed)	C-SC	M-SC (proposed)
Scenario-1	1000	5	> 50	15.85	0.07791	0.02661
Scenario-2	1000	25	> 50	31.85	0.01729	0.01056
Scenario-3	1000	40	> 50	27.45	0.01088	0.005812
Scenario-4	1000	60	> 50	25.1	0.007285	0.003513
Scenario-5	200	30	48.1	27.85	0.01182	0.006781
Scenario-6	200	50	49.65	24.1	0.007395	0.0037
Scenario-7	200	90	49.1	21.65	0.004194	0.001831
Scenario-8	200	120	49.2	24.3	0.003164	0.001321

**Figure 15:**
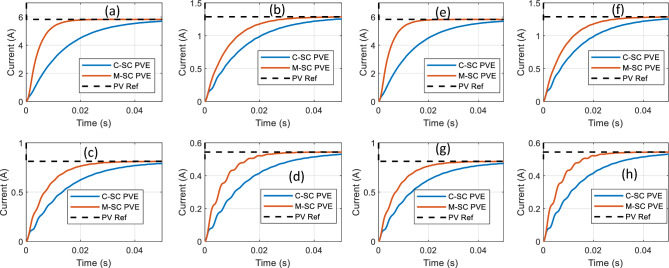
Dynamic response of the PVE in Test profile-1. (**a**) Scenario-1 (**b**) Scenario-2 (**c**) Scenario-3 (**d**) Scenario-4 (**e**) Scenario-5 (**f**) Scenario-6 (**g**) Scenario-7 (**h**) Scenario-8.

The responses of the M-SC and C-SC PVEs for the 8 scenarios is presented graphically in Fig. 15a–h, while the quantitative performance measures are summarized in Table 3. It is consistently seen that the proposed PVE records a rapid transient response, faster than the C-SC. In the first four scenarios, the C-SC records settling time greater than 50 ms, while the M-SC achieves a maximum settling time of just 31.85 ms, which occurs at scenario-2. For the scenarios 5–8, the M-SC further shows an outstanding capability of limiting the setting time, which contributes to its superiority. The C-SC records a minimum settling time of 48.1 ms which occurs at scenario-5 as compared to 27.85 ms for the proposed M-SC in the same scenario. The proposed M-SC therefore becomes approximately two times faster in transient than the C-SC. It is also noted that all the scenario corresponds to the CVR region of the I-V curve. Current control normally has a well behaved response in the CCR. Thus we focus our examinations on the CVR wherein the PVE may be prone to oscillations due to sensitivity of the reference current caused by the voltage. It is therefore seen across the different scenarios that the both PVEs accurately handle the control problem in the CVR since a common I-R model is used for reference current generation. It is also evidenced in Table 3 that the M-SC consistently records the lowest IAE across operation scenarios.

### Test profile 2- continuously varying G, and T

**Figure 16:**
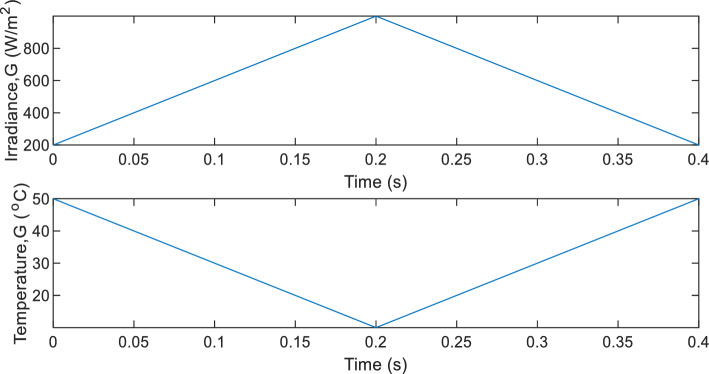
continuously changing G, T in Test profile 2. (Above) Irradiance. (Below) Temperature.

In this test, the PVEs are subjected to continuously changing irradiance and temperature at different load conditions as shown in Fig. 16.

**Figure 17:**
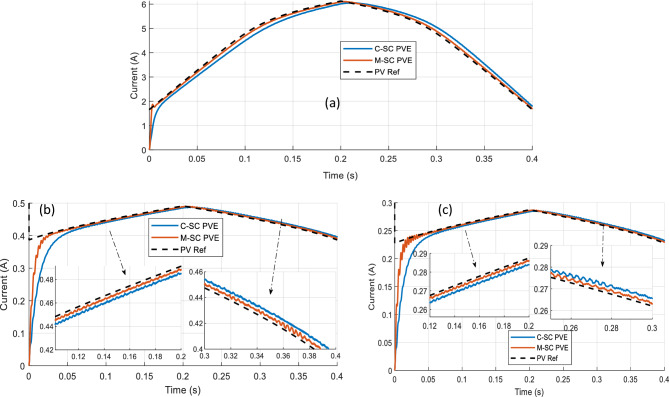
Dynamic response of PVEs under continuously changing G, T, for different ${R}_{pve}$ (**a**) ${R}_{pve}=5\Omega$ (**b**)${R}_{pve}=70\Omega$ (**c**) ${R}_{pve}=120\Omega$.

Three different consistent loads are adopted to have a detail assessment of the PVE. These loads are ${R}_{pve}=5\Omega , 70\Omega , 120\Omega$. For all these operation settings, the dynamic response of the PVEs (current) are plotted on the same graph along with that of the actual PV panel as seen in Fig. 17a–c.

The IAE and emulation efficiency are used to quantitatively assess and compare the PVEs. These results are summarized in Table 4. In the first load condition, it is seen that the proposed PVE clearly outperforms the C-SC as evidenced from its closer alignment with the actual PV. This attributed to its faster dynamic response (see Fig. 17a). A similar scenario is observed when ${R}_{pve}=70\Omega$ and 120Ω as seen in Fig. 17b,c. Thus the efficiency of the proposed PVE must be higher than that of the C-SC based PVE. Their efficiencies are summarized in Table 4. Both the C-SC and proposed record a worst value of efficiency of approximately 96.42% and 98.6199% occurring at ${R}_{pve}=120\Omega$. This is approximately a 2.2% increase in efficiency contributed by the M-SC. The IAE on the other hand has the maximum value of 0.08584 for the C-SC as compared to the value of 0.02933 recorded at ${R}_{pve}=5\Omega$. This represents an improvement in IAE by approximately 0.0565 or (5.65%).

**Table 4:** Quantitative result of the PVEs under continuously varying Irradiance and Temperature.

Performance index	R_pve_ = 5Ω		R_pve_ = 70Ω		R_pve_ = 120Ω	
	C-SC	M-SC (proposed)	C-SC	M-SC (proposed)	C-SC	M-SC (proposed)
IAE	0.08584	0.02933	0.006984	0.003185	0.004087	0.00167
Efficiency (%)	99.2198	99.8964	96.4502	98.4571	96.4155	98.6199

### Test profile 3- changing load conditions

This teste profile seeks to assess the dynamic performance of the PVE under rapidly changing load. In this realm, the PVEs are subjected to abrupt increase and decrease in load. Change the load introduces some disturbances in the close loop control system. The controller should be robust and fast in maintaining rapid tracking of the reference current in order to preserve a high emulation accuracy.

**Figure 18:**
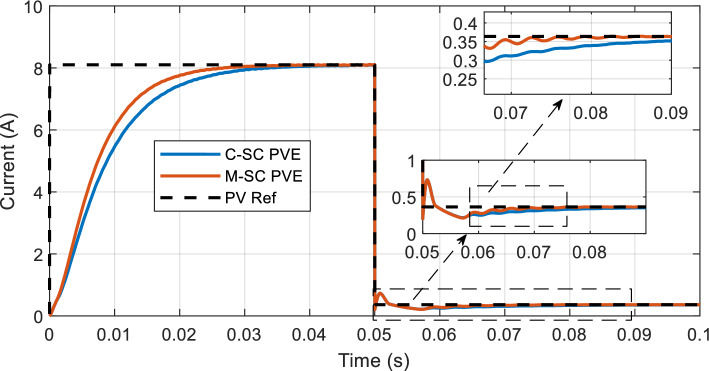
Dynamic response of the PVE: Increasing load from ${R}_{pve}=2\Omega$ to 90Ω.

The response of the PVEs (current) to a an increase load from $2\Omega$ to 90Ω is presented in Fig. 18. It can be seen that both controllers rapidly respond to the disturbance and enforce mirroring of the solar panel through smooth tracking of the reference current. However, the M-SC has a faster response to the abrupt disturbance, recording a recovery settling time of 26.91 ms as compared to the over 50 ms recorded by the C-SC. Furthermore, the M-SC achieves an IAE of 0.06159 as compared to 0.07443 achieved by the C-SC.

**Figure 19:**
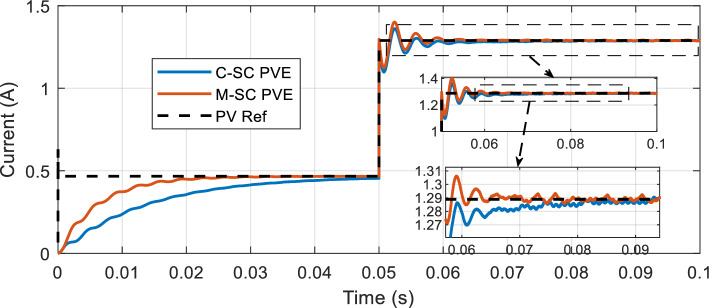
Dynamic response of the PVE: Increasing load from ${R}_{pve}$=70Ω to 25Ω.

A similar scenario is observed when the load abruptly steps down from ${R}_{pve}$=70Ω to 25Ω as seen in Fig. 19, introducing a more intense disturbance in the close loop system. The two controllers undergo oscillations in transient before settling at their steady-state. The proposed controller further imposes is superiority as it recovers from the disturbance in just 18 ms as compared to 31.02 ms for the C-SC. This consistently shows that the proposed modification is a significant improvement of the conventional shift controller.

## Conclusions

In this paper, the issue of precisely and effectively controlling a PVE was examined. A novel control strategy was put forth, which was built upon the conventional shift controller. The objective of the modified shift controller (M-SC) was to modify the control instruction entered into the PVE in response to load conditions. The study demonstrated that the piecewise linear-logarithmic adaptive function under consideration is exceptionally explicit and utilizes measurements from easily accessible sensors. This attribute enhances the overall simplicity of the proposed controller. The control problem pertaining to a 200W PVE was addressed by the controller. A sequence of benchmarked simulations was furnished in order to bolster the analytical construction of the article. Constantly, it has been noted that the integration of the new controller improves the PVE's transient response, accuracy, and efficiency. The proposed controller demonstrated exceptional performance in enhancing the PVE to mirror the efficiency of the actual solar panel under conditions of continuously variable irradiance and temperature, among other test profiles. The significant improvement in emulator operation resulting from the new controller described in this paper has been repeatedly demonstrated in a number of numerical results; thus, experimental prototyping is the main future avenue of this paper.

## Data Availability

The datasets used and/or analysed during the current study available from the corresponding author on reasonable request.
